# A Novel Diagnostic Aid for Detection of Intra-Abdominal Adhesions to the Anterior Abdominal Wall Using Dynamic Magnetic Resonance Imaging

**DOI:** 10.1155/2016/2523768

**Published:** 2016-01-03

**Authors:** David Randall, John Fenner, Richard Gillott, Richard ten Broek, Chema Strik, Paul Spencer, Karna Dev Bardhan

**Affiliations:** ^1^Medical Physics Group, Department of Cardiovascular Science, University of Sheffield, Beech Hill Road, Sheffield S10 2RX, UK; ^2^The Rotherham NHS Foundation Trust, Rotherham Hospital, Moorgate Road, Rotherham S60 2UD, UK; ^3^Radboud University Medical Centre, Department of Surgery, P.O. Box 9101, 6500 HB Nijmegen, Netherlands

## Abstract

*Introduction*. Abdominal adhesions can cause serious morbidity and complicate subsequent operations. Their diagnosis is often one of exclusion due to a lack of a reliable, non-invasive diagnostic technique. Development and testing of a candidate technique are described below.* Method*. During respiration, smooth visceral sliding motion occurs between the abdominal contents and the walls of the abdominal cavity. We describe a technique involving image segmentation and registration to calculate shear as an analogue for visceral slide based on the tracking of structures throughout the respiratory cycle. The presence of an adhesion is attributed to a resistance to visceral slide resulting in a discernible reduction in shear. The abdominal movement due to respiration is captured in sagittal dynamic MR images.* Results*. Clinical images were selected for analysis, including a patient with a surgically confirmed adhesion. Discernible reduction in shear was observed at the location of the adhesion while a consistent, gradually changing shear was observed in the healthy volunteers.* Conclusion*. The technique and its validation show encouraging results for adhesion detection but a larger study is now required to confirm its potential.

## 1. Introduction

Abdominal adhesions are pathological formations of fibrous scar tissue that tether or adhere abdominal structures. As a complication of abdominal surgery they may be the cause of serious morbidity and may complicate subsequent operations. A combination of non-specific symptoms and an aversion to unnecessary surgery leads to a conservative patient management strategy that often fails to tackle the underlying condition. Surgical procedures (laparoscopy, laparotomy) are currently the only reliable way to determine if a patient has adhesions, but such intervention may induce further adhesions. A non-invasive diagnostic technique would therefore be invaluable for effective patient management and reducing surgical complications.

During the respiratory cycle the abdominal contents slide smoothly against the confines of the abdominal cavity (abdominal wall, etc.)—a process termed visceral slide. Although absence of, or disturbance to, visceral slide is considered an indicator of adhesions, the literature contains very few quantitative attempts at visceral slide measurement [1–3]. The use of dynamic MR for adhesion detection has had reported success but examination of the images in sufficient detail to detect abnormal slide has proven labour intensive and results are subject to high inter-operator variability [4–6]. We have previously presented a technique to mathematically analyse movement within the whole of the abdomen to help infer the presence of gross abnormalities (extensive adhesions) [6]. This current paper outlines a refinement of this technique using image segmentation and registration to exclusively interrogate more subtle abnormalities on the abdominal wall by examination of visceral slide.

Image registration is a mathematical process which aims to warp points in one image to match their corresponding points in another. It has a proven value in tracking features or structures between incrementally varying images. However, sliding geometry (such as in the abdomen) is recognised to challenge registration algorithms [7–11]. To address this issue the literature has largely focused on development of highly sophisticated, bespoke registration algorithms to accurately account for sliding [7–11]. In this paper our focus is different: we intend to evaluate the sliding motion itself. We consider that there is benefit in using “off-the-shelf” registration technology combined with a protocol optimised for shear detection, and for this purpose we promote a segmentation-registration method. Such a pragmatic approach makes the technique more transparent and the technology more accessible, hopefully encouraging clinical adoption.

To the authors' knowledge nobody has accomplished quantitative characterisation/measurement of the sliding motion in the abdomen nor has a reliable technique been developed for non-invasive abdominal adhesion detection. With this in mind this paper is a “work in progress” that communicates an overview of the methodology developed and presents preliminary results.

## 2. Method

Our scanning protocol was developed independently which led to a protocol that echoed that of Lienemann et al. (2000) [4]. Dynamic MR images are acquired using a True FISP (true fast imaging with steady-state precession) MR imaging sequence. Images are obtained in the sagittal plane from the mid ascending colon to mid descending colon, which covers the full extent of the abdominal contents. Scanning parameters include a matrix size of 256 × 256, a slice thickness of 7 mm, and 10 mm gaps between slices. 30 frames are acquired at each sagittal slice location with an approximate time between frames of 0.4 seconds. Patients are scanned in the supine position and asked to bear down and breathe normally during the acquisition of each sagittal slice (for ~12 seconds) capturing approximately 3 respiratory cycles.

**Figure 1:**
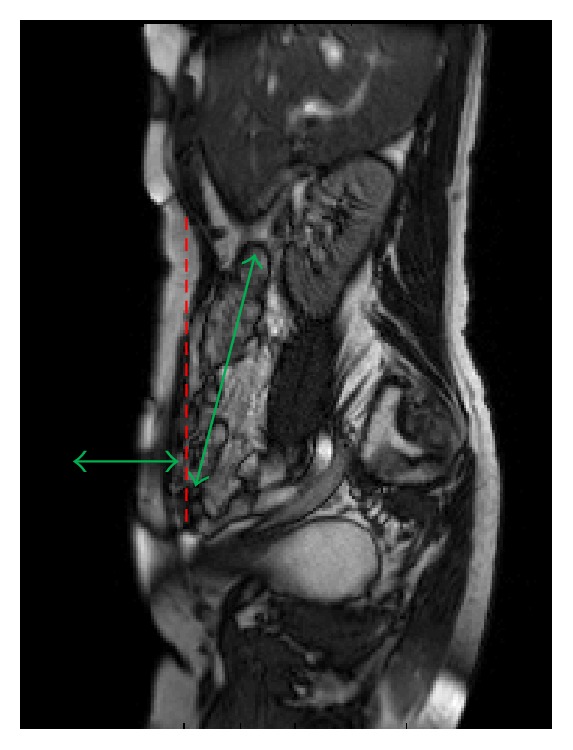
Schematic of the motion discontinuity in the abdomen during respiration. The horizontal green arrow indicates the predominant motion of the abdominal wall whilst the mostly vertical arrow represents the predominant motion of the abdominal contents. The dotted red line indicates the approximate location of the motion discontinuity.

The focus of our method is a particular sliding motion system, characterised as one in which two adjacent structures in contact slide independently against each other. A schematic of the type of motion observed in the abdomen during respiration is shown in Figure 1.

These types of systems involve a discontinuity in the motion along the boundary separating the two moving objects. The method aims to determine the degree of sliding by quantifying shear as an analogue for the sliding motion taking place at the discontinuity. The amount of shear refers to the difference in the relative displacement of the two objects on either side of the motion discontinuity along the boundary.

**Figure 2:**
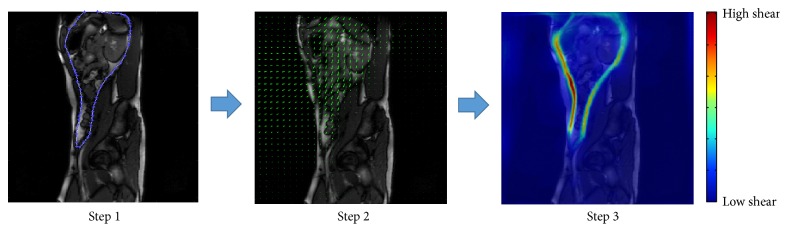
Flow chart describing the methodology. Step  1: typical region drawn to separate (segment) the two regions of different motion; step 2: depiction of the mathematically quantified movement; step 3: depiction of the shear taking place along the boundary in a “sheargram”.

The method relies on a segmentation step that requires that the boundary between the two regions of motion be defined, as shown in step 1 of Figure 2. This is done semi-automatically by manually defining the boundary on a single frame, after which the position of the boundary is tracked for all subsequent frames. The motion within the two regions can now be mathematically interrogated separately without interference from one another. Separate registrations quantify the motion in each region which are then recombined to reconstruct a full description of motion over the whole image. The motion is depicted as arrows (vectors) in step 2 of Figure 2. The relative motions along the boundary over the whole dynamic image sequence are then computed to determine the amount of shear. The result is a “sheargram”: the coloured band in step 3 of Figure 2 depicting the total shear along the boundary over approximately 3 respiratory cycles.

## 3. Results

**Figure 3:**
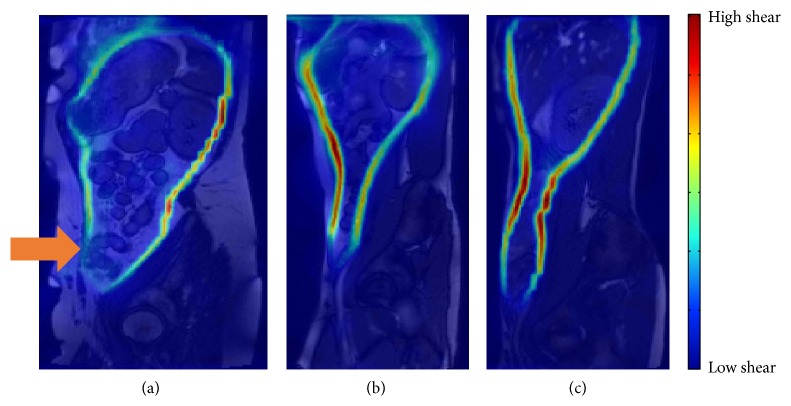
Comparison of the sheargrams from (a) a patient with an adhesion (arrow) and (b) and (c) two healthy volunteers.

For the purposes of this exercise we obtained a selection of suitable MR images in which complementary surgical confirmation was available to clarify the degree of adhesive pathology. Of particular interest was a patient with a surgically confirmed adhesion to the anterior abdominal wall following a hernia repair. The result of the shear summed over approximately 3 respiratory cycles for this patient is compared to two healthy volunteers without adhesions in Figure 3.

An apparent reduction in shear is observed at the site of the surgically confirmed adhesion (highlighted by the arrow) which contrasts with the relatively uniform, gradually changing shear observed along the abdominal wall of the two healthy volunteers.

## 4. Validation

A critical assessment of our method demands evidence that the technique is robust and bereft of artefacts. In the absence of a clinical trial or a pilot study this section discusses two examples of validation tests, with interpretation of results and implications for clinical use. Validation tests that have been performed to assess the robustness of the technique include:

1. A highly idealised computer-generated stretching of a rectangular region of an MR image
2. Imaging of a physical system involving the compression of a sponge in a syringe to generate a sliding against the syringe wall.

### 4.1. Test 1

**Figure 4:**
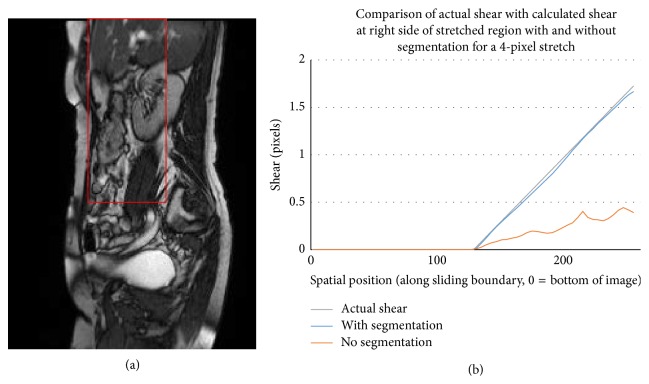
Validation experiment 1 with an idealised stretch of the portion of a MR image shown in (a) and shear results compared to actual shear in the system in (b).

A rectangular section of an abdominal MR image was artificially stretched relative to the surrounding MR image (shown in Figure 4(a)) to create a movie of discontinuous sliding with known, time-dependent shear at the boundary. The shear along the boundary was calculated with and without the segmentation step and compared to the known shear along the boundary in Figure 4(b).

The shear calculated when motion segmentation is included closely matched the known shear at the boundary of the stretched section. The largest discrepancy occurred at the top of the image (see Figure 4(b)) and is attributable to detail being stretched outside the image space. Even with the relatively small shears present in this example the measured shear agreed within approximately 5% of the actual shear. The simple nature of the deformation (uniform stretch) does not challenge the registration algorithm but it does demonstrate the inherent accuracy of the procedure in the absence of  “real-world” complexities.

### 4.2. Test 2

**Figure 5:**
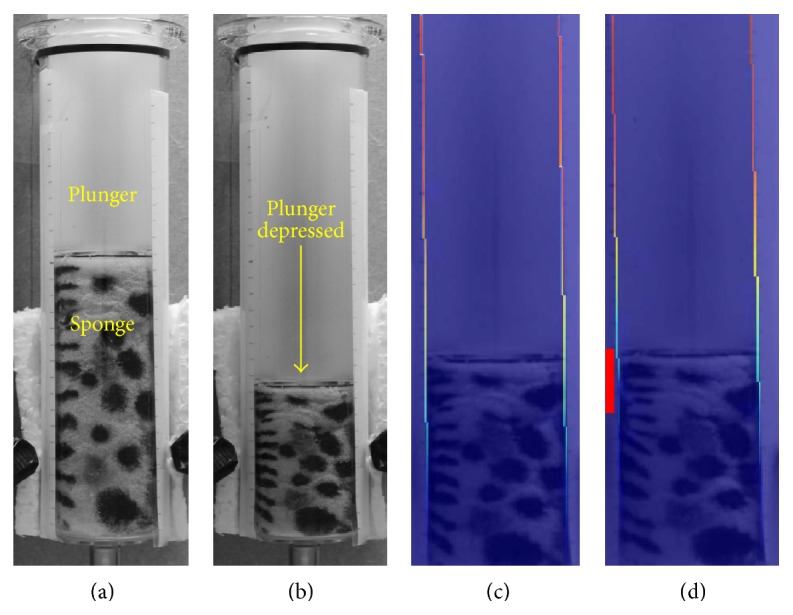
Syringe test object displaying (a) uncompressed sponge, (b) compressed sponge, (c) shear result without adhesive tape, and (d) shear result with adhesive tape (indicated by red block).

The second validation test was physical rather than computationally simulated and involved the compression of a textured sponge within a syringe (Figure 5). The plunger was used to gradually compress the sponge while images were taken with a standard DSLR camera (Cannon EOS 1100D). Two separate sets of acquisitions were made: in the first, the sponge was allowed to be freely compressed; for the second, an adhesive piece of double sided sticky tape was added to the inside of the syringe to create a localised resistance to the sponge's “motion” thereby disrupting slide (an analogue for an adhesion). The images in Figures 5(a) and 5(b) show the uncompressed and compressed sponge while the images in Figures 5(c) and 5(d) used our segmentation-registration protocol to depict the shear summed over the whole compression with and without the presence of the adhesive tape. This test offers a more realistic challenge for the algorithm as it includes non-uniform deformation and localised variations in sliding motion. It not only assesses the technique's ability to quantify shear but also its ability to detect an adhesive area along the boundary—proof of principle for adhesion detection.

When qualitatively observing the sponge's motion unaided by the sheargram, determining the location of the adhesion was extremely challenging. When combined with the images in Figures 5(c) and 5(d), a sufficient reduction in shear around the location of the adhesion was observed to accurately raise awareness of its presence.

## 5. Discussion

Intra-abdominal adhesions can form anywhere in the abdomen, vary in shape and size, and therefore cause a spectrum of symptoms: little or none at one end to severe, frequent pain at the other. A proportion of patients with adhesions are forced to repeatedly seek medical attention for their unexplained abdominal pain. In current clinical practice a patient with severe abdominal pain and suspected bowel obstruction will undergo non-invasive imaging [12–15]. Planar X-ray, fluoroscopy, CT or MRI may be used in an attempt to detect a proximal region of distended bowel with an abrupt reduction in bowel calibre to a collapsed distal region [13]. Importantly, the radiological features determine the site of obstruction but not necessarily the cause: an adhesion may be likely but not proven. The only definitive method to prove the presence of adhesions is by surgery (laparotomy or laparoscopy) which itself is often the primary cause of adhesions [16]. As a result they place a significant burden on healthcare worldwide [16–18] and the lack of a reliable non-invasive diagnostic technique results in conservative patient management and prolonged patient discomfort [12].

It is recognised that improved diagnostic methods are required to reliably inform patient management strategies for adhesive bowel obstruction [12] but additionally we propose a requirement for diagnosis of adhesions in symptomatic patients* without* intestinal obstruction. A potential diagnostic technique is radiological examination of cine-MRI to observe the motion of the abdominal contents. This was first described by Lienemann et al. in 2000 [4] and has led to several further publications [5, 19, 20] from the same group. The cine-MRI acquisition acquires slices in the transverse and sagittal planes and requires a radiologist to identify regions of absence of movement which could correspond to adhesive pathology. The technique has shown promise and reported impressive accuracies, identifying up to 89% of surgically confirmed adhesions [19].

However, in our experience, radiological assessment of cine-MR images is limited by its difficulty, high inter-operator variability, and excessive reporting time. These factors led to our previous publication which described mathematical mapping and depiction of movement in the abdomen to aid the radiologist [6]. This current paper offers a refinement to our previous approach by presenting shear measurements as a diagnostic metric for the presence and location of more subtle adhesive pathologies around the perimeter of the abdominal cavity. The measurement of shear could be used to influence decisions on whether to operate, facilitate more efficient surgery due to improved adhesion localisation, and reduce the risk of serious surgical complications such as bowel perforation during incisions.

### 5.1. Non-Clinical Validation

The validation tests were idealised and non-clinical but permitted the analysis method to be verified, offering a proof of principle for the detection of adhesive regions. The result of Test 1 (stretched MRI region) showed a close match between the output of the computer analysis and actual shear, indicating correct shear calculation. This was echoed equally well in the less idealised experiment of Test 2 (textured sponge). Although a small amount of shear was observed at the site of the adhesion (see Figure 5), this was visibly attributable to the weakness in bonding between tape and sponge as a small amount of slippage occurred. A more subtle observation is the reduction in shear on the opposite wall to the adhered region in Figure 5(d) when compared to the acquisition without an adhesion in Figure 5(c). Close examination of the images and registration deformation field confirm that this is not a failing in the shear analysis but rather the adhesion influenced the deformation at the right-hand boundary as well as the left. With the region below the adhesion remaining largely uncompressed some sponge moved laterally into this space rather than sliding vertically downward against the syringe wall.

The results of both tests offer support for the technique showing that it accurately captured shear and that this could be used to detect an area disturbed by an adhesive influence.

### 5.2. Clinical Test

Application to a handful of clinical examples has thus far continued to produce promising results. In the case reported here reduced shear was observed at the site of a surgically confirmed adhesion while in a sample of healthy abdominal scans (*n* = 4) a smoother more gradual change in shear was observed. The combined evidence of the clinical outcome and the validation tests provides reassurance that the technique has merit. Developing our system for clinical use requires two major steps: retrospective application to a larger patient cohort with surgical confirmation and a prospective programme.

The clinical results in Figure 3 also reveal areas of reduced shear which do not correspond to a confirmed adhesion (e.g., upper left Figure 3(a) and at the very base of the abdomen in all images). Inspection of movement in these areas reveals that this is not a failure of the technique to measure shear correctly but rather confirms that sliding is genuinely reduced in these areas. At this stage of development the aim of this technique is not to provide a standalone diagnostic outcome but to draw the eye of the radiologist toward specific suspect areas, which when combined with other diagnostic information can enable an informed decision to be made. This initial extra investment by the radiologist is potentially more than offset by increased accuracy of diagnosis and reduction in examination time. It is likely that there will be common sites of shear reduction which, with experience, should be easily identified and interpreted appropriately. A future ambition is the production of a shear “atlas” to provide a typical map of shear in health and disease to help clarify such issues.

### 5.3. Challenges and Future Work

This paper has reported on a work in progress and there remain challenges which must be addressed before the proposed diagnostic protocol for anterior wall adhesions can be considered reliable. The principal concerns relate to (i) sensitivity of the results to position of boundary placement between the moving regions and (ii) possible artefacts introduced by structures moving through the 2D imaging plane. With reference to (i), our experience confirms that the placement of the boundary is relatively consistent due to high contrast anatomy; consequently reproducible results are achievable. With respect to (ii), through plane motion in 2D is most effectively addressed by 3D imaging. However, advantages gained from the 2D implementation are the high temporal resolution not available in 3D imaging and the simplicity and speed of implementation. Also, notably, movement within the abdomen is mostly superior-inferior; therefore objects largely remain in the sagittal imaging plane. It is for these reasons that complementary 2D and 3D analyses are being pursued.

As a final comment, the protocol is intentionally designed to support the use of different “off-the-shelf” registration algorithms. Currently the majority of work has been performed using the Sheffield Image Registration Toolkit (ShIRT) but ANTs (Advanced Normalisation Toolkit, an open source registration algorithm) has also been successfully incorporated and used.

## 6. Conclusion

A technique to measure shear to infer the amount of visceral slide along the extremities of the abdominal cavity has been proposed, investigated, and validated. Despite the acknowledged limitations of the current implementation, the preliminary results have shown the adopted methodology to be successful in determining and detecting the locations of adhesions. Clinical application is currently limited by the small number of patients examined but an additional study is being pursued with a larger cohort of patients for further assessment.
